# Thermocatalytic CO_2_ Conversion over a Nickel-Loaded Ceria Nanostructured Catalyst: A NAP-XPS Study

**DOI:** 10.3390/ma14040711

**Published:** 2021-02-03

**Authors:** Adrián Barroso-Bogeat, Ginesa Blanco, Juan José Pérez-Sagasti, Carlos Escudero, Eric Pellegrin, Facundo C. Herrera, José María Pintado

**Affiliations:** 1 Department of Materials Science and Metallurgical Engineering and Inorganic Chemistry, Faculty of Sciences, University of Cádiz, Campus Río San Pedro s/n, 11510 Puerto Real (Cádiz), Spain; josemaria.pintado@uca.es; 2 Institute for Research in Electron Microscopy and Materials (IMEYMAT), Faculty of Sciences, University of Cádiz, Campus Río San Pedro s/n, 11510 Puerto Real (Cádiz), Spain; 3 Central Services of Scientific and Technological Research (SC-ICYT), University of Cádiz, Campus Río San Pedro s/n, 11510 Puerto Real (Cádiz), Spain; juanjose.perez@uca.es; 4 ALBA Synchrotron Light Source, Carrer de la Llum 2-26, 08290 Cerdanyola del Vallès (Barcelona), Spain; cescudero@cells.es (C.E.); eric.pellegrin@zeiss.com (E.P.); herrera.facundo@gmail.com (F.C.H.); 5 Institute for Theoretical and Applied Physicochemical Research (INIFTA, CONICET), Department of Chemistry, Faculty of Exact Sciences, National University of La Plata, Diagonal 113 and 64, La Plata 1900, Argentina

**Keywords:** ceria nanocubes, nickel, CO_2_ hydrogenation, rare earth oxides, Near Ambient Pressure X-ray Photoelectron Spectroscopy (NAP-XPS)

## Abstract

Despite the increasing economic incentives and environmental advantages associated to their substitution, carbon-rich fossil fuels are expected to remain as the dominant worldwide source of energy through at least the next two decades and perhaps later. Therefore, both the control and reduction of CO_2_ emissions have become environmental issues of major concern and big challenges for the international scientific community. Among the proposed strategies to achieve these goals, conversion of CO_2_ by its reduction into high added value products, such as methane or syngas, has been widely agreed to be the most attractive from the environmental and economic points of view. In the present work, thermocatalytic reduction of CO_2_ with H_2_ was studied over a nanostructured ceria-supported nickel catalyst. Ceria nanocubes were employed as support, while the nickel phase was supported by means a surfactant-free controlled chemical precipitation method. The resulting nanocatalyst was characterized in terms of its physicochemical properties, with special attention paid to both surface basicity and reducibility. The nanocatalyst was studied during CO_2_ reduction by means of Near Ambient Pressure X-ray Photoelectron Spectroscopy (NAP-XPS). Two different catalytic behaviors were observed depending on the reaction temperature. At low temperature, with both Ce and Ni in an oxidized state, CH_4_ formation was observed, whereas at high temperature above 500 °C, the reverse water gas shift reaction became dominant, with CO and H_2_O being the main products. NAP-XPS was revealed as a powerful tool to study the behavior of this nanostructured catalyst under reaction conditions.

## 1. Introduction

Since the start of the Industrial Revolution in 1750, carbon-rich fossil fuels (i.e., oil, coal, and natural gas) have become essential raw materials for the production not only of energy but also of commodity chemicals [1]. Despite the obvious environmental benefits and increasing governments’ economic incentives associated with their substitution, these fuels are foreseen to remain as the dominant primary energy source in the medium-term future [2]. Such an extensive use, together with the still low efficiency of the vast majority of energetic processes, has been causing a steady rise in the atmospheric levels of CO_2_, the major human-related greenhouse gas, during the past two centuries and especially in the second half of the 20th century [1,3].

One of the possible strategies to reduce atmospheric CO_2_ levels entails its conversion in high added value products, such as fuels or chemicals [4,5,6]. CO_2_ reduction with H_2_ is an interesting way to address this important challenge, as it can use hydrogen obtained from water electrolysis by employing the electricity coming from renewable sources [7,8,9,10]. CO_2_ hydrogenation can lead to the production of CO, methanol, or hydrocarbons depending on the catalyst and reaction conditions [11,12]. Reverse water–gas shift reaction (rWGS) is another way to obtain CO [13], which can be later used as feedstock in other processes, for example, to obtain fuels. More intense research efforts are still required in order to make these reactions sustainable in large-scale processes [11], and a great deal of work has been done in testing metal/reducible oxides catalysts. Although pathways for these reactions are not yet clear, there is a certain agreement concerning the way both the supported metal phase and the oxide are involved in the reaction. In this regard, H_2_ activation is performed by the supported metal, whereas the support provides oxygen vacancies to activate CO_2_ [14,15,16].

Cerium oxide has be considered an excellent candidate as a reducible support for CO_2_ activation due to its well-known basic properties [17], as well as to its ability to create surface oxygen vacancies when being reduced [18]. Concerning the metal, nickel has been revealed as a good choice due to its low price, relative stability, and good properties for reactions involving CO_2_ [19,20,21,22,23]. However, one of the main drawbacks when using nickel is related to its sintering and formation of undesirable carbon deposits when working under harsh conditions [24,25]. Both sintering and coke resistance can be improved if nickel is combined with a lanthanoid oxide such as ceria [26].

Recently, our research group successfully prepared and characterized ceria nanocubes with enhanced reducibility as compared to bulk ceria [26]. Herein, this nanostructured ceria has been used as support for nickel nanoparticles, as a possible combination that is expected to give good results in the CO_2_ hydrogenation reaction.

To study this catalyst, Near Ambient Pressure X-ray Photoelectron Spectroscopy (NAP-XPS) was chosen, as it can provide information in quasi-real conditions. This technique is becoming a great tool to study catalysts’ performance in conditions close to real ones [27].

## 2. Materials and Methods

### 2.1. Synthesis of the Nickel-Loaded Ceria Nanocubes Catalyst

The synthesis of the Ni-loaded CeO_2_ NCs catalyst sample was carried out in 2 consecutive stages using simple surfactant-free wet chemistry methods. First, CeO_2_ NCs with an average edge length of around 50 nm were prepared by a facile hydrothermal procedure, which had been previously reported elsewhere [28] and successfully applied by our research group to the synthesis of both pure [29] and Ln-doped CeO_2_ NCs, Ln representing the lanthanoid elements La [30,31] and Pr [32]. In brief, 125 mL of a 17.3 mol·L^−1^ NaOH (99%, Scharlau, Sentmenat, Spain) aqueous solution, 100 mL of a 0.12 mol·L^−1^ Ce(NO_3_)_3_·6H_2_O (99.5%, Alfa Aesar, Kandel, Germany) aqueous solution, and 15 mL of deionized water were mixed in a polypropylene beaker and magnetically stirred for 30 min at room temperature. The resulting grayish homogeneous suspension was poured into a 250 mL Teflon container, which was placed in a stainless-steel autoclave reactor and tightly sealed. This reactor was heated at 180 °C for 24 h in an electric oven. After this time had elapsed, the system was allowed to slowly cool down to room temperature, and then the yellowish solid product was separated by centrifugation, thoroughly washed several times with deionized water until neutral pH of the liquid phase and washed once with absolute ethanol (VWR Chemicals, Briare, France), and finally oven-dried at 80 °C for 24 h. This synthetic procedure yielded about 1.9 g of CeO_2_ NCs (i.e., approximately 92% overall yield), so it was performed in duplicate to obtain the required mass of product for later tasks, including its physicochemical characterization and support of the nickel catalytic phase.

In a second stage, a 5 wt.% nickel nominal loading was supported on the as-prepared CeO_2_ NCs by employing a surfactant-free controlled chemical precipitation method based on a slight modification of a previous method also developed by our research group [33,34,35]. Ni(NO_3_)_2_ and hexamethylenetetramine (HMT, hereafter), which are readily soluble both in water and ethanol, were selected as precursor and precipitant agent, respectively. Then, 1 g of the freshly synthesized CeO_2_ NCs and an excess amount of HMT (99%, Scharlab), equivalent to a HMT/Ni^2+^ molar ratio of 5, were dispersed in 60 mL of ethanol (96%, VWR Chemicals, France) with the assistance of ultrasonication. The suspension was heated from room temperature up to 75 °C under continuous mechanical stirring of 800 rpm and using a reflux tube cooled with water to avoid solvent losses. Once the system reached this temperature, 20 mL of an aqueous solution containing the appropriate mass of Ni(NO_3_)_2_·6H_2_O (99.9%, Sigma Aldrich, Steinheim, Germany) for achieving the desired metal loading in the final multicomponent catalyst was slowly added to the suspension at a rate of 0.33 mL·min^−1^ by a highly precise syringe pump (KD Scientific, Holliston, MA, USA). The reaction mixture was additionally aged for 1 h under identical heating and stirring conditions. After this time, the obtained precipitate was centrifuged, repeatedly washed with deionized water to eliminate any remnants of reagents and washed once with ethanol, and oven-dried at 80 °C overnight. Finally, the oven-dried solid was ground, sieved, and calcined in a muffle furnace with a heating rate of 5 °C·min^−1^ up to 370 °C and kept at such temperature for 4 h. The resulting powder catalyst sample will be henceforth denoted as “5Ni-CeO_2_ NCs.”

### 2.2. Physicochemical Characterisation

#### 2.2.1. Chemical Composition

The actual nickel loading of the freshly prepared nanocatalyst sample was estimated by X-ray fluorescence (XRF) analysis carried out in a M4 Tornado energy dispersive spectrometer from Bruker (Billerica, MA, USA), equipped with Mo Kα radiation (λ = 0.7107 Å) source operating at 50 kV and 600 μA. The nickel content of the 5Ni-CeO_2_ NCs sample, as determined by XRF, was around 5 wt.%, corresponding to 13.3 at.%.

#### 2.2.2. Structural Characterization

X-ray diffraction (XRD) patterns for both the CeO_2_ NCs support and the fresh Ni catalyst were collected at room temperature in a D8 ADVANCE diffractometer from Bruker, operating with Cu Kα radiation (λ = 1.5406 Å), and under the following specific acquisition conditions: 2*θ* range from 10 to 90°, step size of 0.02°, and step counting time of 38.4 s.

#### 2.2.3. Specific Surface Area

The specific surface area (*S*_BET_) of the nanomaterials was assessed by applying the Brunauer, Emmet, and Teller equation [36] to their respective N_2_ adsorption-desorption isotherms in the relative pressure (*p*/*p*^o^) range between 0.05 and 0.20. These isotherms were registered at −196 °C using an automatic Autosorb iQ_3_ equipment (Quantachrome, Boynton Beach, FL, USA). Prior to starting the adsorption-desorption measurements, about 100 mg of each powder sample was out-gassed under vacuum at 200 °C for 4 h in order to remove moisture and any possible gases and vapors from the laboratory atmosphere adsorbed on the materials surface.

With this technique, a specific surface area of around 30 m^2^·g^−1^ was measured for both samples.

#### 2.2.4. Electron Microscopy Characterization

The freshly prepared nanomaterials were characterized by means of advanced electron microscopy techniques, such as high-resolution transmission electron microscopy (HRTEM), high-angle annular dark field-scanning transmission electron microscopy (HAADF-STEM), and energy-dispersive X-ray spectroscopy (X-EDS). These studies were accomplished in a Talos F200X scanning transmission electron microscope (FEI, Thermo Scientific, Waltham, MA, USA), coupled to a X-EDS ChemiSTEM system (Thermo Scientific) implementing four windowless SDD detectors [37,38]. The powdered samples, as prepared without any further treatment, were mounted on holey carbon-coated TEM grids. Processing of X-EDS data was performed by the Thermo Scientific Velox software (version 2.13).

#### 2.2.5. Reducibility Measurements

Characterization of the redox behavior of both the pristine CeO_2_ NCs and the 5Ni-CeO_2_ NCs catalyst was accomplished by the temperature-programmed reduction (TPR) technique, followed by mass spectrometry (MS). TPR-MS studies were performed in a setup equipped with a quadrupole mass spectrometer (Thermostar GSD301T1, Pfeiffer Vacuum, Wetzlar, Germany) to accurately monitor the composition of the outlet gas stream. Around 100 mg of sample was used in each of these experiments. Prior to starting the experiments, both samples were submitted to cleaning pretreatment: consisting of oxidation under a 60 cm^3^·min^-1^ STP flow of O_2_(5%)/He at 350 °C for 1 h, followed by cooling down in the same atmosphere to approximately 150 °C. Then, the gas was changed to He for additional cooling down to room temperature, thus avoiding oxygen adsorption on samples surface. Subsequently, the TPR-MS experiments were carried out in a 60 cm^3^·min^−1^ STP flow of H_2_(5%)/Ar from room temperature to 950 °C at a heating rate of 10 °C·min^−1^. The nanomaterials were kept at this temperature for 1 h. The mass/charge (*m*/*z* henceforward) ratios registered during these runs were 2 (H_2_^+^) to follow hydrogen consumption and 18 (H_2_O^+^) for the associated water evolution. In this connection, it should be pointed out that, for the sake of simplicity, TPR results are plotted as water evolution profiles, since a complete agreement with the corresponding hydrogen consumption curves was found for both nanomaterials. The *m*/*z* ratios 28 (CO^+^), 40 (Ar^+^), and 44 (CO_2_^+^) were also recorded.

#### 2.2.6. Surface Basicity Characterization

Surface basicity (i.e., both the nature and concentration of basic sites) of the raw CeO_2_ NCs and the Ni nanocatalyst sample was studied by temperature-programmed desorption (TPD) of pre-adsorbed CO_2_ followed by mass spectrometry (MS). These TPD-MS diagrams were recorded in the same equipment previously employed for the TPR-MS essays. About 100 mg of powder sample was subjected to the aforementioned standard cleaning pretreatment in O_2_(5%)/He at 350 °C for 1 h. After cooling down to room temperature, the pretreated nanomaterial was exposed to a 60 cm^3^·min^−1^ STP flow of pure CO_2_ (*P*_CO2_ = 1 atm) for 1 h in order to saturate the sample surface with adsorbed CO_2_. Then, the sample was flushed with a flow of 60 cm^3^·min^−1^ STP of pure Ar at room temperature for 1 h to remove the physically adsorbed CO_2_. Finally, the TPD-MS experiment was carried out in the same flow from room temperature up to 900 °C at a heating rate of 10 °C·min^−1^. During this analysis, MS signals for *m*/*z* 12 (C^+^), 28 (CO^+^), 40 (Ar^+^), and 44 (CO_2_^+^) were registered. Additionally, similar TPD-MS experiments were also accomplished by exposing the pretreated oxide samples to the pure CO_2_ stream at 500 °C for 1 h and subsequently cooling down to room temperature under the same atmosphere.

Furthermore, additional information regarding the surface basicity of the prepared nanomaterials was derived from CO_2_ volumetric chemisorption experiments. The CO_2_ isotherms were also recorded on the Micromeritics ASAP 2020 apparatus and employing around 100 mg of sample. Once again, prior to the adsorption measurements, the materials were pretreated in a O_2_(5%)/He atmosphere at 350 °C for 1 h, followed by 1 h evacuation at the same temperature and under a residual pressure below 10^−6^ Torr. Then, 2 consecutive CO_2_ isotherms were acquired at 35 °C and over the partial pressure range from 0 to 1013 mbar, with an evacuation treatment at identical temperature for 1 h in between them.

#### 2.2.7. XPS and NAP-XPS Measurements

Conventional XPS (X-ray Photoelectron Spectrocopy) measurements were performed in a Kratos Axis Ultra^DLD^ spectrometer (Kratos Analytical Ltd., Manchester, UK), using monochromatized Al Kα (*hν* = 1486.6 eV), and X-ray power of 150 W. High-resolution spectra were acquired with a pass energy of 20 eV under the Fixed Analyzer Transmission (FAT) mode. Samples were pressed into self-supported pellets and fixed by means of a conductive double-sided carbon polymer tape. Charging effects were compensated with the coaxial charge neutralizer device developed by Kratos, and the binding energy (BE) scale was corrected with respect to adventitious carbon and set to 284.8 eV [39].

Near Ambient Pressure XPS (NAP-XPS) measurements were performed in CIRCE beamline, ALBA Synchrotron Light Source, Barcelona, Spain. Samples were pressed into pellets over a gold mesh which was used to minimize charging effects during the experiments. For each core level, acquisition was performed with 2 different mean kinetic energies (KE), namely KE = 550 eV and KE = 190 eV. Table 1 summarizes the different photon energies used for the acquisition of the different core levels. Using the QUASES-IMFP-TPP2M software, Ver. 3.0, developed by S.Tougaard (©2016) (QUASES-Tougaard Inc., Odense, Denmark), the IMFP was calculated [40], and the approximated sampling depth was 3.3 nm for KE = 550 eV and 1.8 nm for KE = 190 eV. This provides the depth profile information of the samples. Prior to any measurement, the samples were submitted to a cleaning treatment under 0.5 mbar O_2_ at 150 °C for 30 min and further cooled to room temperature under the same atmosphere. Then, the samples were successively treated under H_2_ (1 mbar), CO_2_ (1 mbar), and a reaction mixture consisting of 0.2 mbar of CO_2_ and 0.8 mbar of H_2_ (CO_2_:H_2_ ratio 1:4). The tested reaction temperatures were 250 °C, 500 °C, 600 °C, and 640 °C. A quadrupole mass spectrometer coupled to the analysis chamber of the NAP-XPS system was used to follow the reactants and products during the experiments.

Spectra were acquired in the FAT mode, with a pass energy of 10 eV. At each photon energy selected, together with the core level, a survey spectrum was acquired. This survey was used to help in the BE scale correction where C 1*s* could not be used. In this sense, the signal for the Ce 3*d u*’’’ peak at 917.0 eV [41] for energy correction when photon energy was 1440 eV and 1080 eV and the signal for Ce 4*p*_3/2_ at 207.2 eV [42] were used for all other spectra. All data, both from the conventional or the NAP-XPS systems, were processed with CasaXPS software (version 2.3.23PR1.0, Casa Software Ltd., Devon, UK). Details from fitting procedures are given in Supplementary Materials file.

## 3. Results and Discussion

### 3.1. Structural and Textural Characterization

**Figure 1:**
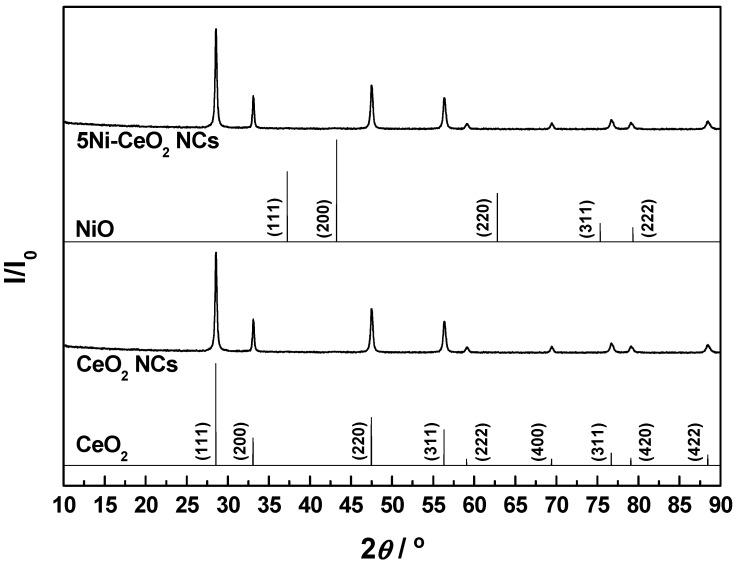
X-ray diffraction (XRD) diagrams recorded for the freshly prepared CeO_2_ nanostructured catalysts (NCs) and 5Ni-CeO_2_ NCs samples. Reference patterns for cubic fluorite-type CeO_2_ and face-centered cubic NiO are also plotted for comparison purposes.

First, a basic structural and textural characterization of the as-prepared CeO_2_ NCs and 5Ni-CeO_2_ NCs samples was performed by XRD and N_2_ physical adsorption, respectively. The recorded XRD diagrams are shown in Figure 1, together with the reference patterns for cubic fluorite-type CeO_2_ (space group *Fm*-3*m*) and face-centered cubic NiO (space group *Fm*-3*m*) for the sake of comparison. As can be seen from the Figure 1, the diffractogram for CeO_2_ NCs was dominated by a well-defined set of very intense and sharp reflection peaks typical of ceria with fluorite structure, which was preserved after nickel incorporation. Furthermore, the absence of clearly distinguishable diffraction peaks ascribable to the cubic NiO phase in the diagram for 5Ni-CeO_2_ NCs advocates for either a relatively high dispersion or an essentially amorphous nature of the supported nickel-containing phases in the sample. More insights into this latter aspect were gained from the electron microscopy study, as discussed below.

**Figure 2:**
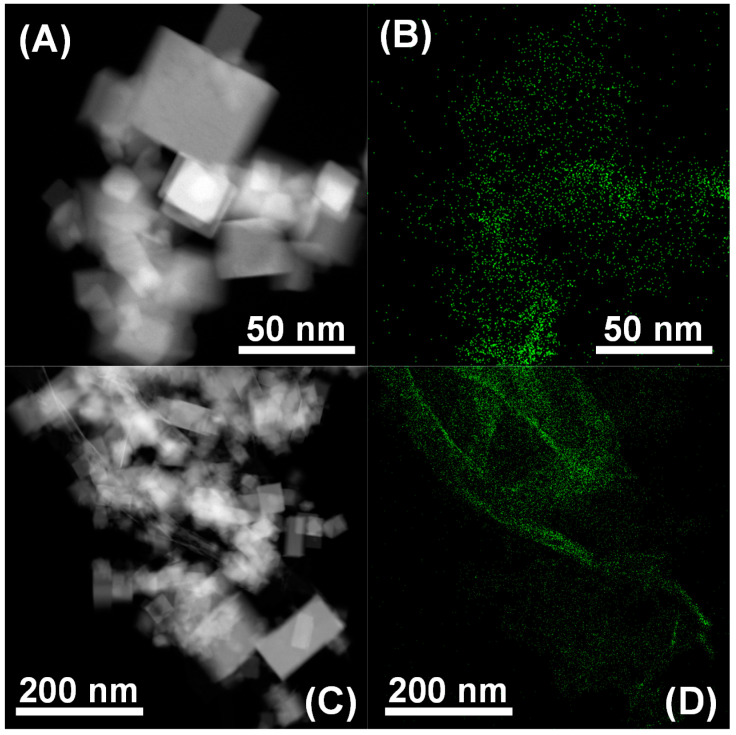
(**A**,**C**) Representative high-angle annular dark field-scanning transmission electron microscopy (HAADF-STEM) images for the freshly prepared 5Ni-CeO_2_ NCs catalyst; (**B**,**D**) the corresponding energy-dispersive X-ray spectroscopy (X-EDS) maps for Ni (green).

Figure 2A,C gathers representative HAADF-STEM images registered for the fresh 5Ni-CeO_2_ nanostructured catalyst. From them, it is clear that this sample in fact consisted of cubic-shaped nanocrystals with edge lengths spanning from 5 mm to nearly 100 nm and an average value of around 50 nm. Furthermore, a HRTEM study (not shown here for the sake of brevity) confirmed that these nanocubes were mostly enclosed by crystallographically well-defined (100) surfaces, with truncations at corners and edges associated with (111) and (110) facets, respectively. This morphological and crystallographic description is basically the same as that obtained for the bare CeO_2_ NCs support (readers are referred to the Supporting Information, Figure S1, for more details), which is also fully consistent with that previously reported by our research group for ceria nanocubes prepared by the same hydrothermal method [29]. Accordingly, neither the morphology nor the nanostructure of the pristine CeO_2_ NCs underwent significant modifications due to the incorporation of the nickel-containing phase by the aforesaid surfactant-free controlled chemical precipitation procedure. Additionally, complementary X-EDS analyses were also carried out on the as-prepared 5Ni-CeO_2_ NCs catalyst in order to confirm the spatial distribution of the first-row transition metal. The resulting maps are displayed in Figure 2B,D, in which the Ni signal appears in green. As can be seen, this chemical element mainly accumulated on the surface of the cubic-shaped ceria nanocrystals in a highly dispersed state, and the presence of large (i.e., up to a few nanometers in size) nickel-based nanoparticles was not detected.

Conventional XPS measurements also confirmed the high dispersion of the nickel-containing nanoparticles in the 5Ni-CeO_2_ NCs catalyst. After registering Ce 3*d*, Ni 2*p*, Ce 4*d*, and Ni 3*p* core levels, the Ni/Ce ratio was estimated at two different depths. Photoelectrons from Ce 3*d* and Ni 2*p* core levels possessed a similar kinetic energy (about 600 eV when using Al K*α* as excitation source), which was different from that of Ce 4*d* and Ni 3*p* (around 1390 eV under the same conditions). These values led to an IMFP close to 1.2 nm for Ce 3*d* and Ni 2*p*, which increased up to 2.2 nm for Ce 4*d* and Ni 3*p* signals. Accordingly, the Ni 2*p*/Ce 3*d* ratio provides information corresponding to a maximum depth of around 3.6 nm from the sample surface (which corresponds to three times the IMFP for these photoelectrons [43]), while the depth of Ni 3*p*/Ce 4*d* increased to 6.6 nm. Ni 2*p*/Ce 3*d* and Ni 3*p*/Ce 4*d* ratios of 0.44 and 0.33, respectively, were obtained from the quantification of the corresponding high-resolution XPS spectra for the fresh nanostructured catalyst. These results are markedly higher than the corresponding Ni/Ce nominal value (i.e., 0.15), thus corroborating the essentially surface character of the nickel-containing phases and their excellent dispersion degree, well in agreement with the conclusions drawn from the XRD and X-EDS analyses.

**Figure 3:**
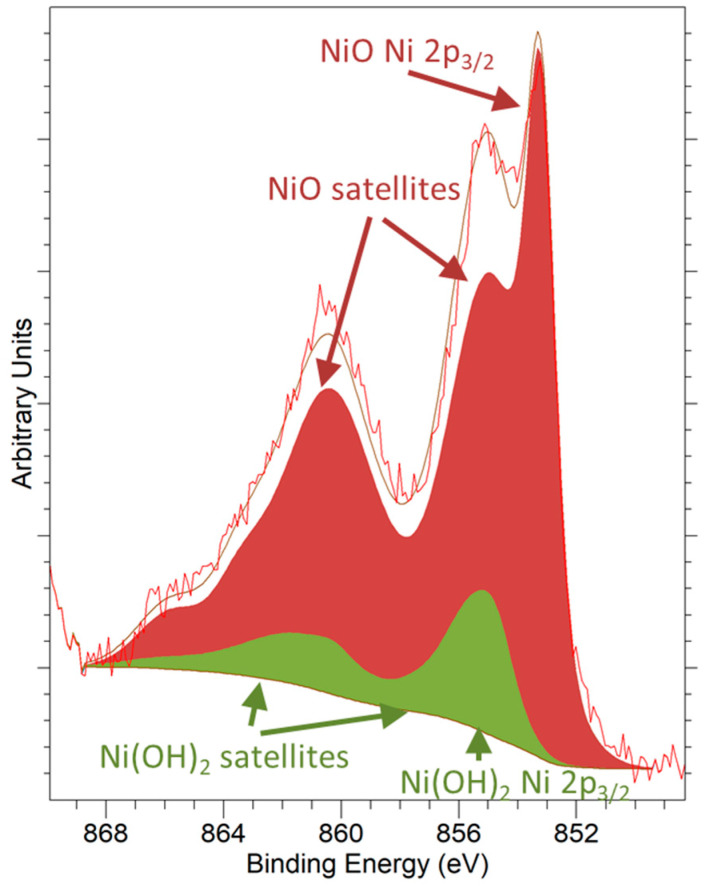
Ni 2*p*_3/2_ XPS signal registered with an Al K*α* X-ray source for the as-prepared 5Ni-CeO_2_ NCs catalyst. Peak decomposition was accomplished using data from Biesinger et al. [44]. Peaks corresponding to NiO and Ni(OH)_2_ appear in dark red and green, respectively (see Supplementary Materials for details on the fitting procedure, Figure S2).

Concerning the chemical state of nickel in the freshly prepared nanostructured catalyst sample, an in-depth analysis and deconvolution of the Ni 2*p*_3/2_ core level in Figure 3 reveals that it was only present in oxidized state, chiefly as NiO (85%), with a small amount of Ni(OH)_2_ (15%). This latter compound was likely formed as a result of chemisorption of water from the laboratory atmosphere during the handling and storage of the calcined sample. Ni 2*p*_3/2_ spectral fitting parameters were extracted from Reference [44] (see Supplementary Materials for details).

### 3.2. Redox Characterization

**Figure 4:**
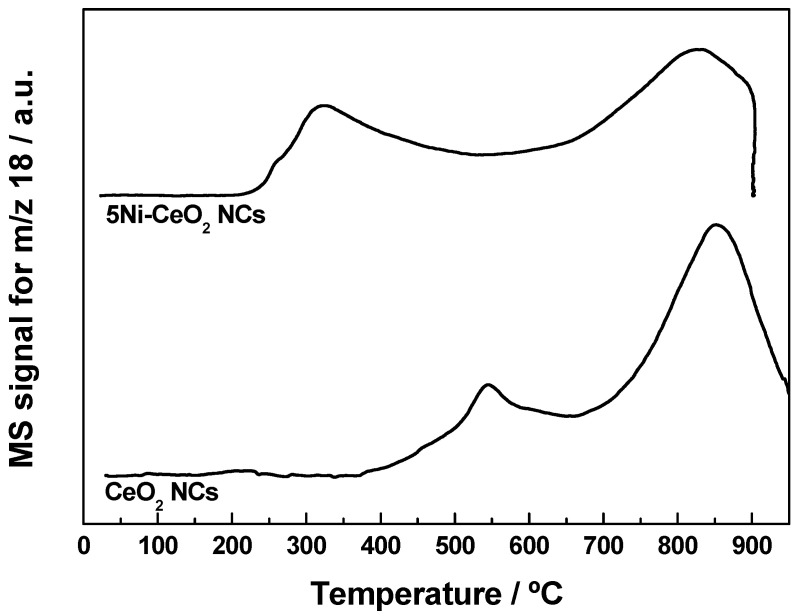
TPR-MS profiles for the CeO_2_ NCs and 5Ni-CeO_2_ NCs samples.

Cerium and nickel oxidation states are well known to play pivotal roles in the interaction with CO_2_, and thereby in the hydrogenation of this chemically stable molecule [6,45,46,47]. In this regard, a number of works have revealed the ability of ceria to activate the CO_2_ molecule by an oxygen-vacancy assisted mechanism [45], which obviously requires the presence of Ce^3+^ cations at the surface level. Moreover, the nickel oxidation state has been tentatively correlated with both the activity and selectivity of ceria-supported nickel catalysts in CO_2_ hydrogenation reactions [6]. Therefore, it is essential to characterize in detail the redox properties of the CeO_2_ NCs and 5Ni-CeO_2_ NCs samples. For such purpose, TPR experiments under flowing H_2_(5%)/Ar were performed on these nanomaterials. The resulting profiles are depicted in Figure 4 as water evolution traces, with a complete consistency with the corresponding hydrogen consumption curves being observed for both samples. Consequently, the different events in the former traces were all unambiguously associated with abstraction of lattice oxygen from the nanomaterials by hydrogen.

As far as the bare CeO_2_ NCs sample is concerned, its profile displays the typical bimodal shape reported in the literature for ceria in the form of nanostructured and high surface area powders [48,49,50,51]. In accordance with the traditional interpretation, the low temperature peak centered at around 550 °C is attributed to surface reduction of CeO_2_, while the much more intense and broader band peaked at 850 °C is connected with bulk oxide reduction. As expected, the incorporation of nickel brought remarkable modifications in the TPR profile of the nanostructured ceria support. Thus, the most prominent change involves the surface reduction feature, which significantly shifted toward much lower temperatures, appearing at about 325 °C, and overlapped with some additional bands, which is likely ascribable to the reduction of the supported Ni^2+^-containing phases (i.e., essentially NiO and Ni(OH)_2_). In addition, the relatively low temperature observed for the reduction of these nickel phases advocates for the presence in the catalyst sample of surface amorphous or very fine reducible species, which weakly interact with the ceria support [52]. Again, these observations are in line with the results derived from XRD, electron microscopy, and conventional XPS analyses. Finally, from the whole set of TPR results, it is clear that the 5Ni-CeO_2_ NCs catalyst exhibited an enhanced reducibility at low temperature in comparison with the pristine CeO_2_ NCs support, while the effect on the high temperature reduction was almost negligible.

**Figure 5:**
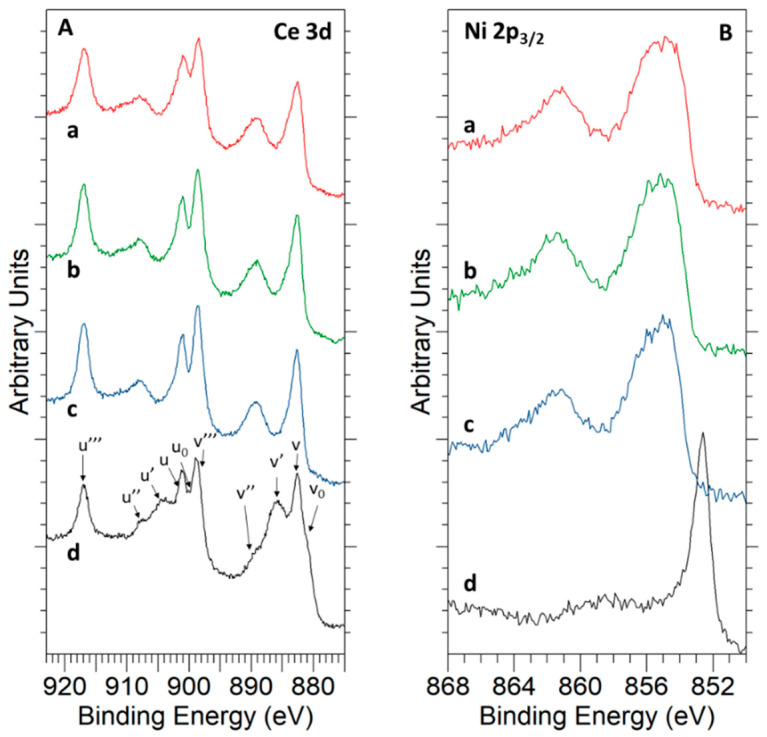
NAP-XPS spectra recorded at KE = 190 eV for Ce 3*d* (**A**) and Ni 2*p*_3/2_ (**B**) core levels of the 5Ni-CeO_2_ NCs catalyst during reduction under hydrogen (1 mbar) at different temperatures: (a) 250 °C, (b) 300 °C, (c) 350 °C, and (d) 500 °C. Ce 3d peaks assignments as proposed by the authors of [53]. For further details of the Ni 2p_3/2_ peak decomposition, see Supplementary Materials File, Figures S5 and S6.

The evolution of the surface chemical features during the reduction process by hydrogen was followed for both samples by means of the NAP-XPS technique. Prior to discussing the obtained results, it should be borne in mind that, during these experiments, the hydrogen pressure (i.e., 1 mbar) was much lower than that in the conventional TPR-MS runs, in which it reached a value of 50 mbar. Accordingly, a marked shift to higher temperatures was expected for the onset of the different reduction events as compared to that observed in the TPR profiles. Table 2 compiles the evolution of ceria reduction degree with the application of different treatments. These values were calculated from NAP-XPS data at the selected analysis depths, i.e., 3.3 nm (KE = 550 eV) and 1.8 nm (KE = 190 eV). Ce^3+^ percentages were estimated as described in Reference [30]. As can be seen from Table 2, cerium reduction was almost negligible for both nanomaterials at temperatures below 500 °C, regardless of the composition of the atmosphere (see Figures S3 and S4 in Supplementary Material). Reduction begins at this temperature, with the Ce^3+^ content being slightly higher for the nickel catalyst sample. It is also worth noting that Ce^3+^ and foreseeable oxygen vacancies were essentially concentrated in the outermost surface layers of the nanocubes. Furthermore, it should be also noted that no ceria reduction was observed at surface level until Ni^2+^ was completely reduced to metallic nickel, as inferred from Figure 5 in which Ce 3*d* and Ni 2*p*_3/2_ core levels were registered under 1 mbar H_2_ and at KE = 190 eV (i.e., analysis depth of 1.8 nm).

By contrast, at temperatures of 500 °C and above, Ce^3+^ fraction is high for both samples, and nickel was found in a completely reduced state, i.e., as Ni(0), irrespective of the atmosphere. In this connection, it is worth highlighting that, although the nickel reduction degree did not change up to a temperature of 350 °C, an evolution of the NiO/Ni(OH)_2_ ratio was clearly identified. Figure S3 depicts the deconvolution of the Ni 2*p*_3/2_ signal for different reduction temperatures and both analysis depths. The relative amount of Ni(OH)_2_ increased from around 20% at 3.3 nm up to 40% at 1.8 nm, suggesting that, as expected, this phase was mainly located at the uppermost layers of the nickel-containing nanoparticles.

An undesirable effect observed during reduction of the catalyst sample was the sintering of nickel-containing particles associated with their reduction to the metallic state. In this regard, it is well known that supported nickel catalysts usually suffer from strong sintering when operating under demanding reaction conditions [24]. As deduced from Table 3, the Ni/Ce ratio strongly decreased after reduction to 500 °C and approached the nominal value of 0.15, thus corroborating the sintering of the nickel nanoparticles under such reducing conditions.

### 3.3. Surface Basicity and Interaction with the CO_2_ Molecule

Ceria is a moderately basic oxide [54,55,56], so it is expected to easily interact with the CO_2_ molecule. Accordingly, this section is devoted to study both the amount and nature of the adsorbed CO_2_ species by means of Temperature Programmed Desorption (TPD) of pre-adsorbed CO_2_ at room temperature, CO_2_ volumetric chemisorption experiments, and NAP-XPS under 1 mbar of pure CO_2_.

**Figure 6:**
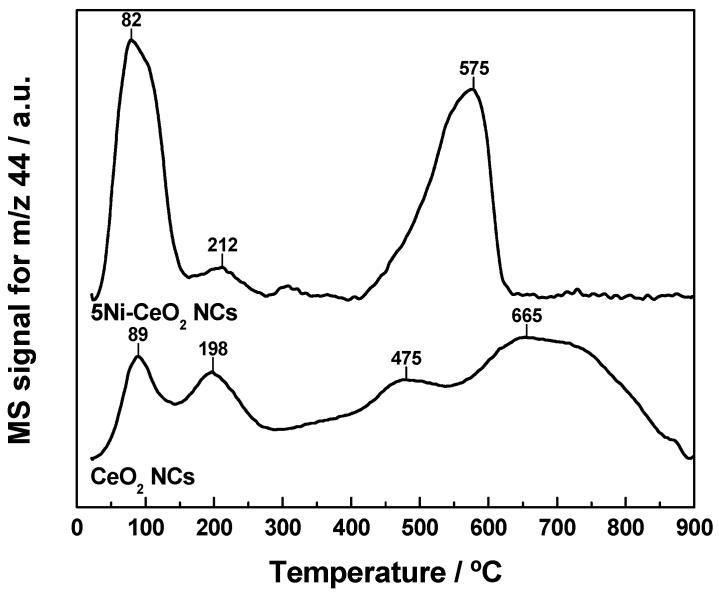
TPD-MS study of CO_2_ (*m*/*z* = 44) pre-adsorbed at room temperature on CeO_2_ NCs and 5Ni-CeO_2_ NCs sample.

Obtained results from the TPD-MS experiments after CO_2_ adsorption at room temperature are displayed in Figure 6. In this regard, it should be kept in mind that the samples were subjected to a cleaning pretreatment in O_2_(5%)/He at 350 °C. Then, they were exposed to the CO_2_ flow at 25 °C for 1 h and finally flushed with Ar at the same temperature for 1 h. Therefore, since this purging removes physically adsorbed CO_2_ species, it is evident that profiles shown in Figure 6 mainly account for those irreversibly adsorbed CO_2_ forms.

Two clearly distinguishable regions can be identified in the TPD-CO_2_ traces. CO_2_ evolution at temperatures above 400 °C is essentially attributable to the thermal decomposition of strongly bound bulk carbonate species, which probably remained in the nanomaterials after applying the cleaning routine. For the purpose of the present study, the low temperature region is much more interesting. As can be seen, the TPD profiles were as a rule dominated by two overlapping major desorption peaks located at about 90 °C and 200 °C. The former was much more intense for the 5Ni-CeO_2_ NCs sample and appeared to result from the overlap of at least two very close and sharp peaks. This CO_2_ evolution feature is likely ascribed to the desorption of weakly bound CO_2_ forms, one of them likely related to NiO, which seems to play a key role in the adsorption of this acidic molecule. The second desorption peak, centered at around 200 °C, is rather similarly shaped for both nanostructured materials and exclusively attributable to CO_2_ adsorption on ceria surface.

Quantitative data for CO_2_ adsorption were obtained from volumetric chemisorption measurements. Adsorbed quantities at room temperature after dosing 7 mbar and 1013 mbar of CO_2_ are collected in Table 4 for both samples. In good agreement with the corresponding TPD profiles, the volumetric data fully confirm the positive effect of the supported nickel phases on the CO_2_ adsorption capability of the bare CeO_2_ NCs support.

Summarizing the results from the TPD-MS and volumetric adsorption studies, the 5Ni-CeO_2_ NCs catalyst sample was able to adsorb larger amounts of CO_2_ than the raw CeO_2_ NCs support both at low and high CO_2_ pressures. Moreover, the interaction of the CO_2_ molecule with the sample surface appeared to be slightly weaker for the nickel catalyst, which makes it a suitable candidate for its application in CO_2_ hydrogenation reactions.

**Figure 7:**
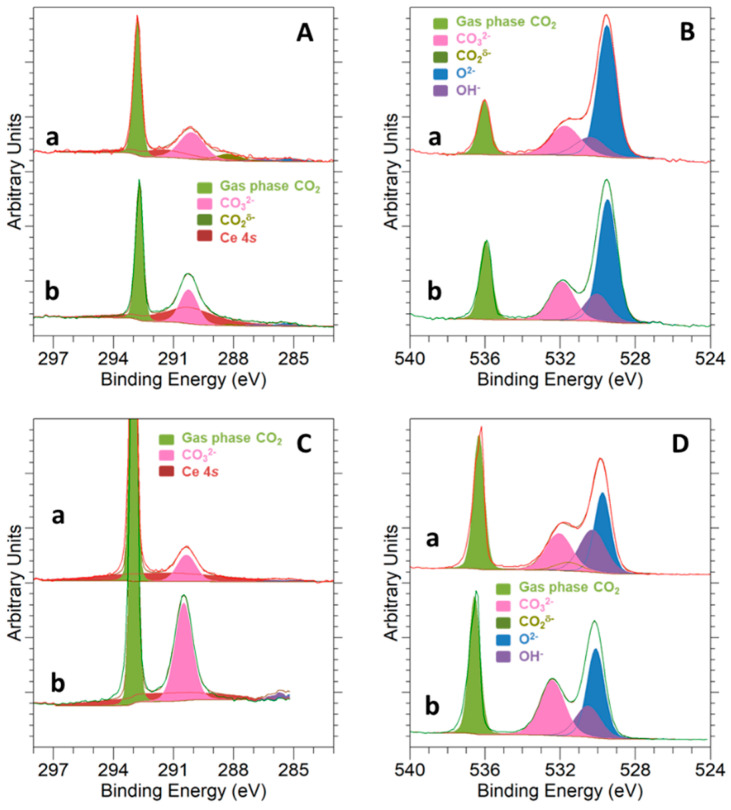
NAP-XPS spectra for C 1*s* at KE = 550 eV and KE = 190 eV (**A**,**C**) and O 1*s* at KE = 550 eV and KE = 190 eV (**B**,**D**) corresponding to (a) 5Ni-CeO_2_ NCs and (b) CeO_2_ NCs under 1 mbar CO_2_ at a temperature of 250 °C.

On the other hand, the interaction of CO_2_ with the samples surface at a temperature of 250 °C was studied by NAP-XPS, with the results displayed in Figure 7. Peaks deconvolution was accomplished as follows: Light green peaks at the higher BE side of C 1*s* and O 1*s* correspond to CO_2_ in the gas phase [57]; pink peaks at about 290.2 eV for C 1*s* and about 532.0 eV for O 1*s* are associated with the carbonate (CO_3_^2−^) species [57]; adventitious carbon components are too small to be significant; blue and purple peaks in O 1*s* spectra are ascribed to the oxide (CeO_2_ and NiO) and OH^-^ groups, respectively; a small dark green peak at about 288.1 eV in the C 1*s* spectrum is related to carboxylate (CO_2_^δ−^) species [57]; and, finally, the broad peak in C 1*s* appearing in dark red corresponds to the Ce 4*s* signal. Details for peak fitting of O 1*s* and C 1*s* signals can be found in Supplementary Materials file, Table S1.

As deduced from Figure 7, carbonate species were the main states for CO_2_ adsorption in both samples, with the corresponding pink peaks being clearly visible in the C 1*s* and O 1*s* spectra. Carbonates were the only CO_2_ adsorption form identified for the bare CeO_2_ NCs, whereas in the case of 5Ni-CeO_2_ NCs, a small number of carboxylates was additionally detected in the C 1*s* spectrum. In this regard, it is worth noting that the identification of carboxylate species in the O 1*s* spectrum is a rather complex task, since their peak is usually centered at BE values very close to those reported for carbonates [57].

**Figure 8:**
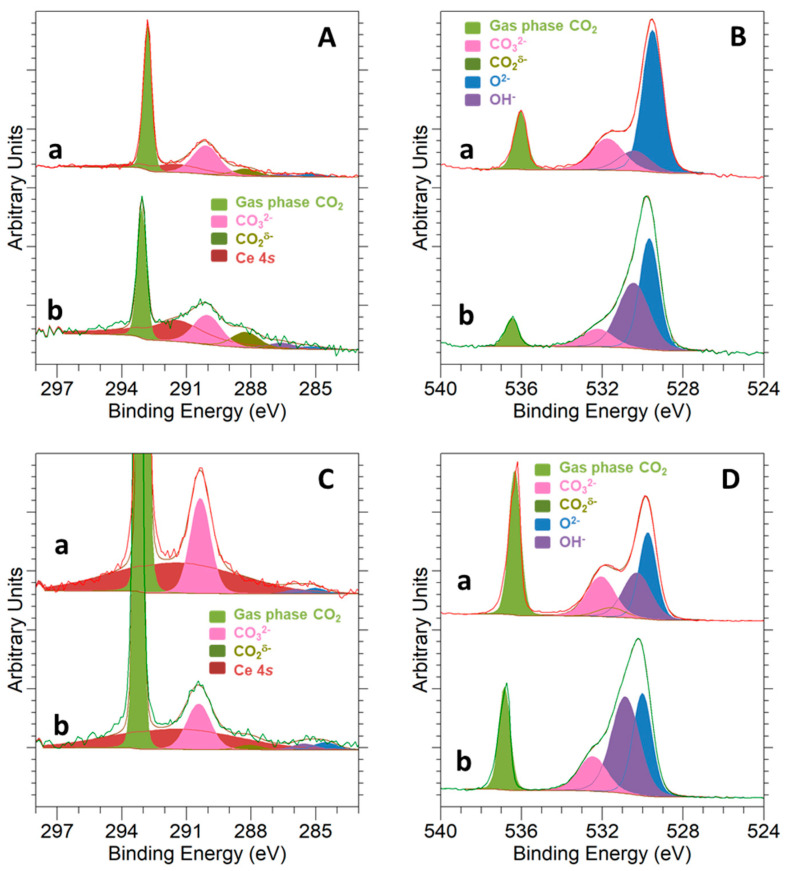
NAP-XPS spectra for C 1*s* at KE = 550 eV and KE = 190 eV (**A**,**C**) and O 1*s* at KE = 550 eV and KE = 190 eV (**B**,**D**) corresponding to 5Ni-CeO_2_ NCs under 1 mbar CO_2_ at temperatures of 250 °C (a) and 500 °C (b).

Finally, NAP-XPS data for CO_2_ adsorption over the 5Ni-CeO_2_ NCs catalyst at two different temperatures, 250 °C and 500 °C, are shown in Figure 8. The peaks deconvolution follows an identical scheme to that previously described for Figure 7. An increase in the temperature for CO_2_ adsorption on the nickel-containing sample led to a decline in the intensity of the carbonate signals, both in the C 1*s* and O 1*s* spectra. Simultaneously, the presence of carboxylate species was more evident in the C 1*s* signals. Furthermore, some noticeable changes concerning the relative intensity of oxide and hydroxyl group peaks in the O 1*s* spectra are also noted. In fact, the intensity of this latter signal increased in comparison with that for the former peak, mainly due to the hydrogen incorporation in the reduction treatment that was applied prior to CO_2_ exposure.

### 3.4. CO_2_ Hydrogenation Reaction Followed by NAP-XPS

The performance of the 5Ni-CeO_2_ NCs catalyst in the CO_2_ hydrogenation reaction was followed in situ by the NAP-XPS technique. The total pressure in the analysis chamber during the catalytic tests was set to 1 mbar, with a gaseous mixture of pure CO_2_ and H_2_ in a molar ratio of 1:4 (i.e., 0.2 mbar CO_2_ and 0.8 mbar H_2_). Starting from 250 °C, the reaction temperature was increased stepwise to 500 °C, 600 °C, and 640 °C, with the latter being the highest temperature that could be reached in the experimental setup. With a view to carefully identifying and following the possible reaction intermediates appearing on the catalyst surface during the reaction, this study was performed at KE = 190 eV to enhance the surface contribution.

Before discussing the obtained NAP-XPS results, it is worth recalling that CO_2_ hydrogenation over supported nickel catalysts is well known to exclusively yield CH_4_ and/or CO as products [11,58]. Therefore, the formation of both oxygenates and long-chain hydrocarbons during the catalytic tests can be completely ruled out. This hypothesis is further corroborated from a careful and detailed analysis of the mass spectrometry signals recorded for different representative m/z ratios during the CO_2_ hydrogenation experiments. From them, only CH_4_ and CO were detected, while no clear evidence of the presence of other reaction products was found.

**Figure 9:**
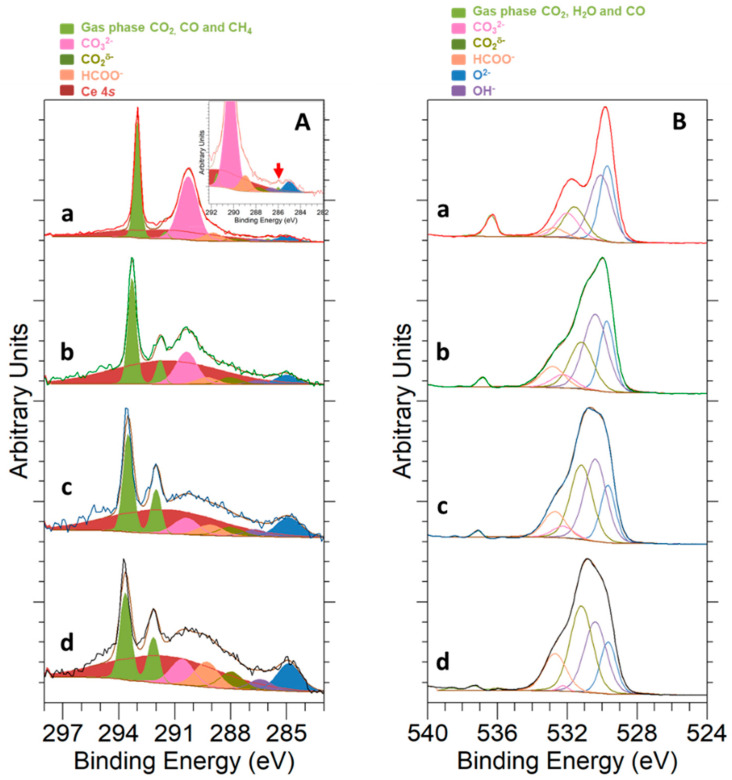
NAP-XPS experiments at KE = 190 eV for C 1*s* (**A**) and O 1*s* (**B**) corresponding to 5Ni-CeO_2_ NCs under reaction conditions (0.2 mbar CO_2_ + 0.8 mbar H_2_) at temperatures of 250 °C (a), 500 °C (b), 600 °C (c), and 640 °C (d). A detail of the C 1*s* signal featuring the peak ascribable to CH_4_ is depicted as an inset.

Figure 9 summarizes the NAP-XPS results registered for the C 1*s* and O 1*s* core levels, which were employed to follow the evolution of the reaction at different temperatures. The color caption for the peaks deconvolution is the same as previously described for Figure 7 and Figure 8. Nevertheless, in this case, we added an additional contribution in orange, which was associated with formate species (HCOO^-^, a typical intermediate in CO_2_ hydrogenation reactions) and set at 289.2 eV and 532.8 eV in the C 1*s* and O 1*s* spectra, respectively [57].

C 1*s* spectra (Figure 9A) reveal a change in reaction selectivity from 250 to 500 °C. A number of possible reaction pathways have been described in the literature for CO_2_ hydrogenation, with each of them leading to different products, such as CO, methanol, and methane [11], depending on the metal active phase and reaction conditions (i.e., temperature and CO_2_:H_2_ molar ratio). At a temperature of 250 °C, the reaction yielded a small amount of methane, which could be detected over the catalyst sample as seen in the inset in Figure 9A. Nonetheless, methane was not identified at higher temperatures, which is well in agreement with the reaction thermodynamics. Instead of this hydrocarbon product, a clearly visible peak for gaseous CO at 291.8 eV was observed with increasing reaction temperatures. This fact advocates for the prevalence of the reverse water–gas shift reaction (referred to as rWGS hereinafter) at temperatures above 500 °C. Furthermore, carbonate peaks also decreased in intensity as the reaction temperature rose, and they were replaced by those ascribed to formate and carboxylate species, which became progressively more relevant in the overall spectra. According to the literature, these formate species, which were absence at low reaction temperatures, are involved as intermediates in the reaction mechanism for rWGS [11].

As far as the O 1*s* high resolution spectra are concerned (see Figure 9B), a gradual increase of the relative intensity of the hydroxyl group peak (colored in purple) with respect to oxide peaks (blue-colored) was noted as the catalyst surface was covered with hydrogen due to increasing temperature. These hydroxyl groups are also involved in different proposed pathways for CO_2_ hydrogenation to methane and CO [11]. On the other hand, the progressive decrease of carbonate species observed in the C 1*s* spectra was also followed from the evolution of the O 1*s* signal. An increase in the reaction temperature from 250 °C to 640 °C was accompanied by a decline in the relative intensity of the carbonates peak from around 13% of the total oxygen detected at 250 °C to only 1% at 640 °C. Finally, both carboxylate and formate intermediates were easily detected in the O 1s profile, increasing their relative intensity as the rWGS progressed and became dominant.

**Figure 10:**
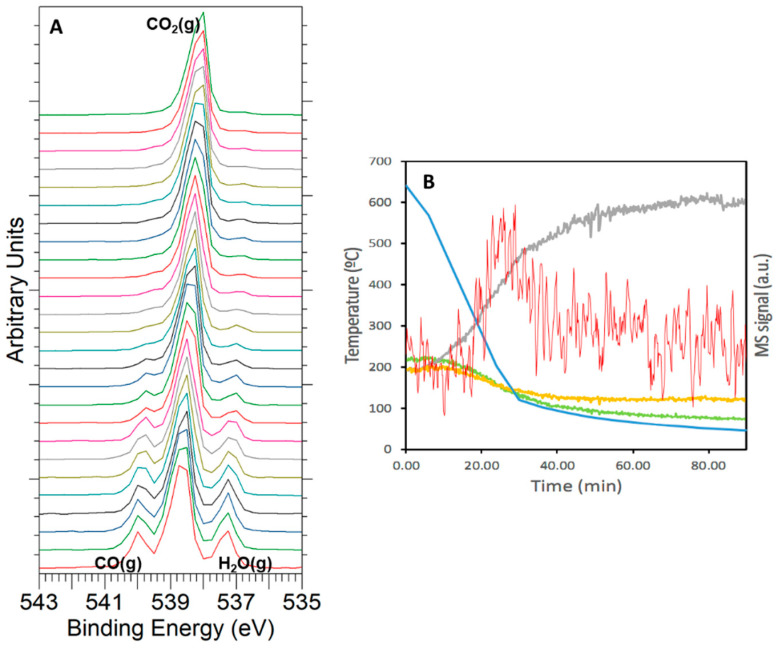
(**A**) Evolution of the O 1*s* signal for the gas phase under reaction conditions when cooling from 640 °C (bottom) to 80 °C (top). (**B**) Mass spectrometry (MS) signal registered for CO_2_ (gray, *m*/*z* = 44), CO (yellow, *m*/*z* = 28), and H_2_O (green, *m*/*z* = 18), and CH_4_ (red, *m*/*z* = 15) during cooling (in blue, temperature trace).

From the above NAP-XPS results, it is observed that the rWGS reaction was dominant at a temperature of 640 °C, so that CO_2_ was almost exclusively reduced to CO. After reaching this latter reaction temperature, additional NAP-XPS spectra were also recorded during the cooling up to room temperature under reaction atmosphere. For such purpose, the sample pellet was pulled forward, so only electrons from the gas phase could be detected, and a series of fast scans of the O 1*s* core level was registered to track the changes in the gas composition during cooling. More than 280 fast scans of O 1*s* signal were collected, with 1 in 10 of them being depicted in Figure 10A. Additionally, a mass spectrometer coupled to the analysis chamber allowed us to monitor the gas composition during cooling, with the evolution of CO_2_, CO, H_2_O, and CH_4_ representative signals plotted in Figure 10B. A steady decrease in the signals for both CO and H_2_O, which were the main products of the rWGS reaction, was observed when cooling from 640 °C to 400 °C. A signal attributable to CH_4_ production was detected at this latter temperature. However, methanation reaction coexisted with rWGS (this latter being less important) until catalytic activity completely ceased at around 100 °C.

## 4. Conclusions

CO_2_ hydrogenation reaction was studied over a nanostructured ceria-supported nickel catalyst using Near Ambient Pressure X-ray Photoelectron Spectroscopy (NAP-XPS). The as-prepared nanocatalyst was first characterized in terms of its physicochemical features. This basic characterization study revealed that the ceria support consisted of cubic-shaped nanocrystals enclosed by crystallographically well-defined (100) facets and with and average edge length of around 50 nm, while the nickel-containing phase was found in a highly dispersed state on the nanocubes surface. Both surface basicity and reducibility features of the ceria support, which are well known to play key roles in CO_2_ hydrogenation, were markedly improved as a result of the incorporation of nickel.

NAP-XPS was revealed as a very useful tool to monitor chemical changes at the surface level occurring during the interaction of CO_2_ with the nanostructured catalyst and under reaction conditions. Furthermore, the use of synchrotron radiation also allowed us to study different sampling depths.

Concerning the catalytic performance, two different temperature ranges were distinguished:

1. Below 500 °C, the catalyst behavior was characterized by:
  - Well-dispersed nickel particles over ceria surface.
  - Both cerium and nickel were in an oxidized state (i.e., Ce^4+^ and Ni^2+^), even under reducing conditions.
  - CO_2_ adsorption and reaction with H_2_ proceeded with high carbonate content on the catalyst surface.
  - Only CH_4_ was detected as hydrogenation product, without evidence of any other reaction, such as reverse water–gas shift (rWGS).

2 Above 500 °C, the catalyst evolution was described by:
  - Strong sintering of supported nickel nanoparticles.
  - Both nickel and cerium were reduced (100% Ni^0^ and a large fraction of Ce^3+^).
  - Much lower amounts of carbonate species were detected on the catalyst surface after CO_2_ adsorption and reaction with H_2_.
  - The appearance of new adsorbed species, which were identified as carboxylates and formates.
  - CO_2_ reduction essentially proceeded via a rWGS mechanism, thus yielding CO as main product and without any evidence of CH_4_ formation.

## Figures and Tables

**Table 1:** Photon energies used for the acquisition of the core levels studied in this paper. All energies are in eV.

Core Level	BE Range	*hν* (KE = 190 eV)	*hν* (KE = 550 eV)
Ce 3*d*+Ni 2*p*	850–925	1080	1440
O 1*s*	525–540	720	1080
C 1*s*	281–293	475	835

**Table 2:** Ce^3+^ percentages for the as-prepared samples estimated from Near Ambient Pressure X-ray Photoelectron Spectroscopy (NAP-XPS) experiments under different conditions.

Sample	Treatment	%Ce^3+^ at 3.3 nm	%Ce^3+^ at 1.8 nm
CeO_2_ NCs	1 mbar H_2_ 250 °C	2	2
	1 mbar 1CO_2_:4H_2_ 250 °C	1	3
	1 mbar 1CO_2_:4H_2_ 500 °C	33	52
	1 mbar 1CO_2_:4H_2_ 600 °C	33	52
	1 mbar 1CO_2_:4H_2_ 640 °C	37	58
5Ni-CeO_2_ NCs	1 mbar H_2_ 250 °C	1	1
	1 mbar H_2_ 300 °C	0	2
	1 mbar H_2_ 350 °C	0	3
	1 mbar H_2_ 500 °C	36	50
	1 mbar 1CO_2_:4H_2_ 250 °C	0	3
	1 mbar 1CO_2_:4H_2_ 500 °C	37	52
	1 mbar 1CO_2_:4H_2_ 600 °C	42	57

**Table 3:** Ni 2*p*_3/2_/Ce 3*d* ratio estimated at different depths for the 5Ni-CeO_2_ NCs catalyst sample subjected to a reduction treatment in 1 mbar H_2_ at increasing temperatures.

Temperature	Ni 2*p*_3/2_/Ce 3*d* at 3.3 nm	Ni 2*p*_3/2_/Ce 3*d* at 1.8 nm
250 °C	0.55	0.74
350 °C	0.55	0.70
500 °C	0.20	0.23

**Table 4:** Amounts of CO_2_ adsorbed on both samples at room temperature. Data are expressed in mmol CO_2_·g^−1^.

Sample	7 mbar CO_2_	1013 mbar CO_2_
CeO_2_ NCs	0.048	0.119
5Ni-CeO_2_ NCs	0.063	0.219

## Data Availability

The data presented in this study are available on request from the corresponding author.

## Supplementary Materials

The following are available online at https://www.mdpi.com/1996-1944/14/4/711/s1, Figure S1. Representative HAADF-STEM and HRTEM images for the bare CeO_2_ NCs support. Ni 2p peak fitting section. Figure S2. Ni 2p_3/2_ XPS signal obtained with Al Kα for the as-prepared 5Ni-CeO_2_ NCs catalyst. Figure S3. Ce 3*d* NAP-XPS spectra recorded at KE = 550 eV (A) and KE = 190 eV (B) corresponding to 5Ni-CeO_2_ NCs under different atmospheres at 250 °C: (a) 1 mbar H_2_, (b) 1 mbar CO_2_, and (c) 1 mbar CO_2_ + H_2_ (1:4). Figure S4. Ce 3*d* NAP-XPS spectra recorded at KE = 550 eV (A) and KE = 190 eV (B) corresponding to 5Ni-CeO_2_ NCs under 1 mbar CO_2_ + H_2_ (1:4) at different temperatures: (a) 250 °C, (b) 500 °C, and (c) 600 °C. C 1*s* and O 1*s* peak fitting section. Figure S5. Ni 2*p*_3/2_ NAP-XPS spectra recorded at KE = 550 eV (A) and KE = 190 eV (B) corresponding to 5Ni-CeO_2_ NCs under 1 mbar H_2_ at different temperatures: (a) 250 °C, (b) 300 °C, and (c) 350 °C. Figure S6. Ni 2p_3/2_ obtained at KE = 190 eV for 5Ni-CeO_2_ NCs under 1 mbar H_2_ at 500 °C. Table S1. C 1*s* and O 1*s* peak fitting parameters.

## References

[1] Kondratenko E.V., Mul G., Baltrusaitis J., Larrazábal G.O., Pérez-Ramírez J. (2013). Status and perspectives of CO_2_ conversion into fuels and chemicals by catalytic, photocatalytic and electrocatalytic processes. Energy Environ. Sci..

[2] U.S. Energy Information Administration (2019). International Energy Outlook 2019 with Projections to 2050.

[3] Mikkelsen M., Jørgensen M., Krebs F.C. (2010). The teraton challenge. A review of fixation and transformation of carbon dioxide. Energy Environ. Sci..

[4] Centi G., Perathoner S. (2009). Opportunities and prospects in the chemical recycling of carbon dioxide to fuels. Catal. Today.

[5] Perathoner S., Centi G. (2014). CO_2_ Recycling: A key strategy to introduce green energy in the chemical production chain. ChemSusChem.

[6] Winter L.R., Chen R., Chen X., Chang K., Liu Z., Senanayake S.D., Ebrahim A.M., Chen J.G. (2019). Elucidating the roles of metallic Ni and oxygen vacancies in CO_2_ hydrogenation over Ni/CeO_2_ using isotope exchange and in situ measurements. Appl. Catal. B Environ..

[7] Graves C., Ebbesen S.D., Mogensen M.B., Lackner K.S. (2011). Sustainable hydrocarbon fuels by recycling CO_2_ and H_2_O with renewable or nuclear energy. Renew. Sustain. Energy Rev..

[8] Costentin C., Robert M., Savéant J.-M. (2013). Catalysis of the electrochemical reduction of carbon dioxide. Chem. Soc. Rev..

[9] Centi G., Quadrelli E.A., Perathoner S. (2013). Catalysis for CO_2_ conversion: A key technology for rapid introduction of renewable energy in the value chain of chemical industries. Energy Environ. Sci..

[10] Appel A.M., Bercaw J.E., Bocarsly A.B., Dobbek H., Dubois D.L., Dupuis M., Ferry J.G., Fujita E., Hille R., Kenis P.J.A. (2013). Frontiers, opportunities, and challenges in biochemical and chemical catalysis of CO_2_ fixation. Chem. Rev..

[11] Kattel S., Liu P., Chen J.G. (2017). Tuning selectivity of CO_2_ hydrogenation reactions at the metal/oxide interface. J. Am. Chem. Soc..

[12] Porosoff M.D., Yan B., Chen J.G. (2016). Catalytic reduction of CO_2_ by H_2_ for synthesis of CO, methanol and hydrocarbons: Challenges and opportunities. Energy Environ. Sci..

[13] Daza Y.A., Kuhn J.N. (2016). CO_2_ conversion by reverse water gas shift catalysis: Comparison of catalysts, mechanisms and their consequences for CO_2_ conversion to liquid fuels. RSC Adv..

[14] Chen C.-S., Cheng W.-H., Lin S.-S. (2003). Study of reverse water gas shift reaction by TPD, TPR and CO_2_ hydrogenation over potassium-promoted Cu/SiO_2_ catalyst. Appl. Catal. A Gen..

[15] Wang X., Shi H., Kwak J.H., Szanyi J. (2015). Mechanism of CO_2_ hydrogenation on Pd/Al_2_O_3_ catalysts: Kinetics and transient DRIFTS-MS studies. ACS Catal..

[16] Chen C., Cheng W., Lin S. (2000). Mechanism of CO formation in reverse water–gas shift reaction over Cu/Al_2_O_3_ catalyst. Catal. Lett..

[17] Bernal S., Blanco G., Gatica J.M., Perez-Omil J.A., Pintado J.M., Vidal H., Adachi G., Imanaka N., Kang Z.C. (2004). Chemical reactivity of binary rare earth oxides. Binary Rare Oxides.

[18] Trovarelli A. (1996). Catalytic properties of ceria and CeO_2_-containing materials. Catal. Rev..

[19] Du G., Lim S., Yang Y., Wang C., Pfefferle L., Haller G.L. (2007). Methanation of carbon dioxide on Ni-incorporated MCM-41 catalysts: The influence of catalyst pretreatment and study of steady-state reaction. J. Catal..

[20] Chang F.-W., Kuo M.-S., Tsay M.-T., Hsieh M.-C. (2003). Hydrogenation of CO_2_ over nickel catalysts on rice husk ash-alumina prepared by incipient wetness impregnation. Appl. Catal. A Gen..

[21] Yamasaki M., Komori M., Akiyama E., Habazaki H., Kawashima A., Asami K., Hashimoto K. (1999). CO_2_ methanation catalysts prepared from amorphous Ni–Zr–Sm and Ni–Zr–misch metal alloy precursors. Mater. Sci. Eng. A.

[22] Yamasaki M., Habazaki H., Asami K., Izumiya K., Hashimoto K. (2006). Effect of tetragonal ZrO_2_ on the catalytic activity of Ni/ZrO_2_ catalyst prepared from amorphous Ni–Zr alloys. Catal. Commun..

[23] Lunde P.J., Kester F.L. (1974). Carbon dioxide methanation on a ruthenium catalyst. Ind. Eng. Chem. Process. Des. Dev..

[24] Wang S., Lu G. (1998). (Max). Role of CeO_2_ in Ni/CeO_2_–Al2O_3_ catalysts for carbon dioxide reforming of methane. Appl. Catal. B Environ..

[25] Rahmani S., Rezaei M., Meshkani F. (2014). Preparation of promoted nickel catalysts supported on mesoporous nanocrystalline gamma alumina for carbon dioxide methanation reaction. J. Ind. Eng. Chem..

[26] Ahmad W., Younis M.N., Shawabkeh R., Ahmed S. (2017). Synthesis of lanthanide series (La, Ce, Pr, Eu & Gd) promoted Ni/γ-Al_2_O_3_ catalysts for methanation of CO_2_ at low temperature under atmospheric pressure. Catal. Commun..

[27] Schnadt J., Knudsen J., Johansson N. (2020). Present and new frontiers in materials research by ambient pressure X-ray photoelectron spectroscopy. J. Phys. Condens. Matter.

[28] Mai H.-X., Sun L.-D., Zhang Y.-W., Si R., Feng W., Zhang H.-P., Liu A.H.-C., Yan C.-H. (2005). Shape-selective synthesis and oxygen storage behavior of ceria nanopolyhedra, nanorods, and nanocubes. J. Phys. Chem. B.

[29] Tinoco M., Fernandez-Garcia S., Lopez-Haro M., Hungria A.B., Chen X., Blanco G., Perez-Omil J.A., Collins S.E., Okuno H., Calvino J.J. (2015). Critical influence of nanofaceting on the preparation and performance of supported gold catalysts. ACS Catal..

[30] Fernandez-Garcia S., Jiang L., Tinoco M., Hungria A.B., Han J., Blanco G., Calvino J.J., Chen X. (2015). Enhanced hydroxyl radical scavenging activity by doping lanthanum in ceria nanocubes. J. Phys. Chem. C.

[31] Collins S.M., Fernandez-Garcia S., Calvino J.J., Midgley P.A. (2017). Sub-nanometer surface chemistry and orbital hybridization in lanthanum-doped ceria nano-catalysts revealed by 3D electron microscopy. Sci. Rep..

[32] Jiang L., Fernandez-Garcia S., Tinoco M., Yan Z., Xue Q., Blanco G., Calvino J.J., Hungria A.B., Chen X. (2017). Improved oxidase mimetic activity by praseodymium incorporation into ceria nanocubes. ACS Appl. Mater. Interfaces.

[33] Cabeza I., Souto L.G., Pintado J.M., Pereira C., Freire C., Blanco G. (2014). Influence of ceria distribution on the redox behaviour of nanoparticulated CeO_2_-SiO_2_ systems with application in catalysis. Surf. Interface Anal..

[34] Bogeat A.B., Núñez-Pérez B., Blanco G., Pintado J.M., Hernández-Garrido J.C., Calvino J.J. (2018). Surface and redox characterization of new nanostructured ZrO_2_@CeO_2_ systems with potential catalytic applications. Surf. Interface Anal..

[35] Bogeat A.B., Raposo I.D., Blanco G., Pintado J.M. (2020). Tuning the integration rate of Ce(Ln)O_2_ nanoclusters into nanoparticulated ZrO_2_ Supports: When the cation size matters. Materials.

[36] Brunauer S., Emmett P.H., Teller E. (1938). Adsorption of gases in multimolecular layers. J. Am. Chem. Soc..

[37] Allen L.J., D’Alfonso A.J., Freitag B., Klenov D.O. (2012). Chemical mapping at atomic resolution using energy-dispersive X-ray spectroscopy. MRS Bull..

[38] Sánchez J.J., López-Haro M., Hernández-Garrido J.C., Blanco G., Cauqui M.A., Rodríguez-Izquierdo J.M., Pérez-Omil J.A., Calvino J.J., Yeste M.P. (2019). An atomically efficient, highly stable and redox active Ce_0.5_Tb_0.5_O_x_ (3% mol.)/MgO catalyst for total oxidation of methane. J. Mater. Chem. A.

[39] Barr T.L., Seal S. (1995). Nature of the use of adventitious carbon as a binding energy standard. J. Vac. Sci. Technol. A.

[40] Tanuma S., Powell C.J., Penn D.R. (1993). Calculations of electron inelastic mean free paths (IMFPS). IV. Evaluation of calculated IMFPs and of the predictive IMFP formula TPP-2 for electron energies between 50 and 2000 eV. Surf. Interface Anal..

[41] Holgado J.P., Alvarez R., Munuera G. (2000). Study of CeO_2_ XPS spectra by factor analysis: Reduction of CeO_2_. Appl. Surf. Sci..

[42] Sarma D., Rao C. (1980). XPES studies of oxides of second- and third-row transition metals including rare earths. J. Electron. Spectrosc. Relat. Phenom..

[43] Tanuma S., Powell C.J., Penn D.R. (1994). Calculations of electron inelastic mean free paths. V. Data for 14 organic compounds over the 50-2000 eV range. Surf. Interface Anal..

[44] Biesinger M.C., Payne B.P., Lau L.W.M., Gerson A., Smart R.S.C. (2009). X-ray photoelectron spectroscopic chemical state quantification of mixed nickel metal, oxide and hydroxide systems. Surf. Interface Anal..

[45] Boaro M., Colussi S., Trovarelli A. (2019). Ceria-based materials in hydrogenation and reforming reactions for CO_2_ valorization. Front. Chem..

[46] Ma Y., Gao W., Zhang Z., Zhang S., Tian Z., Liu Y., Ho J.C., Qu Y. (2018). Regulating the surface of nanoceria and its applications in heterogeneous catalysis. Surf. Sci. Rep..

[47] Winter L.R., Gomez E., Yan B., Yao S., Chen J.G. (2018). Tuning Ni-catalyzed CO_2_ hydrogenation selectivity via Ni-ceria support interactions and Ni-Fe bimetallic formation. Appl. Catal. B Environ..

[48] Yao H.C., Yao Y.F.Y. (1984). Ceria in automotive exhaust catalysts: I. Oxygen storage. J. Catal..

[49] Giordano F., Trovarelli A., De Leitenburg C., Giona M. (2000). A model for the temperature-programmed reduction of low and high surface area ceria. J. Catal..

[50] Désaunay T., Bonura G., Chiodo V., Freni S., Couzinié J.-P., Bourgon J., Ringuedé A., Labat F., Adamo C., Cassir M. (2013). Surface-dependent oxidation of H_2_ on CeO_2_ surfaces. J. Catal..

[51] Perrichon V., Laachir A., Bergeret G., Fréty R., Tournayan L., Touret O. (1994). Reduction of cerias with different textures by hydrogen and their reoxidation by oxygen. J. Chem. Soc. Faraday Trans..

[52] Lechkar A., Bogeat A.B., Blanco G., Pintado J.M., El Begrani M.S. (2018). Methanation of carbon dioxide over ceria-praseodymia promoted Ni-alumina catalysts. Influence of metal loading, promoter composition and alumina modifier. Fuel.

[53] Romeo M., Bak K., El Fallah J., Le Normand F., Hilaire L. (1993). XPS study of the reduction of cerium dioxide. Surf. Interface Anal..

[54] Rosynek M.P. (1977). Catalytic properties of rare earth oxides. Catal. Rev..

[55] Bernal S., Blanco G., Calvino J., Omil J.P., Pintado J. (2006). Some major aspects of the chemical behavior of rare earth oxides: An overview. J. Alloys Compd..

[56] Sato S., Takahashi R., Kobune M., Gotoh H. (2009). Basic properties of rare earth oxides. Appl. Catal. A Gen..

[57] Zhong L., Chen D., Zafeiratos S. (2019). A mini review of in situ near-ambient pressure XPS studies on non-noble, late transition metal catalysts. Catal. Sci. Technol..

[58] Vesselli E., Schweicher J., Bundhoo A., Frennet A., Kruse N. (2010). Catalytic CO_2_ hydrogenation on nickel: Novel insight by chemical transient kinetics. J. Phys. Chem. C.

