# Supplementary material for: Thermocatalytic CO2 Conversion over a Nickel-Loaded Ceria Nanostructured Catalyst: A NAP-XPS Study
Source: Materials (Basel). 2021 Feb 3;14(4):711. doi: 10.3390/ma14040711 (PMC7913549; doi:10.3390/ma14040711)
Supplement: Supplementary file 1 [file materials-14-00711-s001.pdf]

materials

MDPI

*Supplementary Material*

# Thermocatalytic $CO_2$ Conversion over a Nickel-Loaded Ceria Nanostructured Catalyst: A NAP-XPS Study

**Adrián Barroso-Bogeat *, Ginesa Blanco *, Juan José Pérez-Sagasti, Carlos Escudero, Eric Pellegrin, Facundo C. Herrera and José María Pintado**

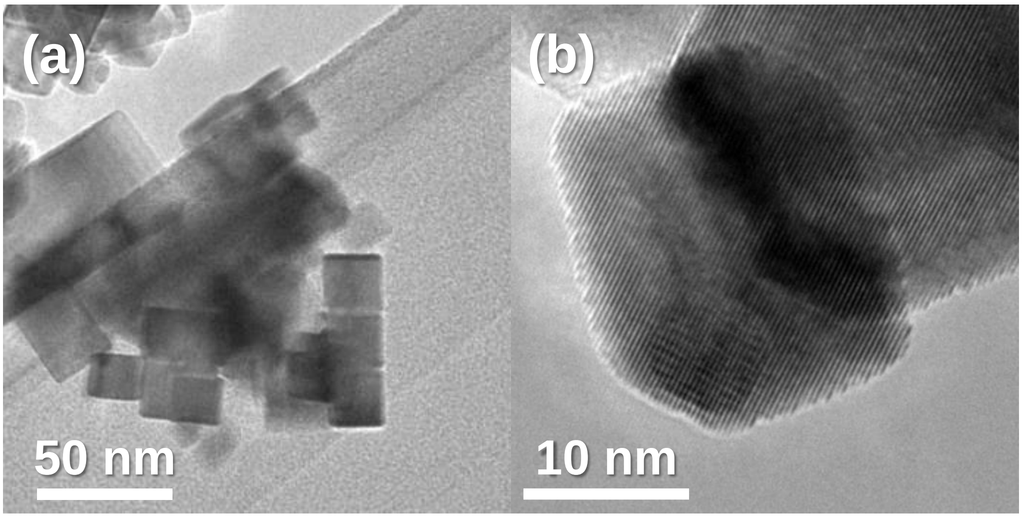


**Figure S1.** Representative (S)TEM images registered for the as-prepared $CeO_2$ NCs sample: (**a**) TEM image at low magnification and (**b**) HRTEM image.

### Ni 2p Peak Fitting

To fit Ni $2p_{3/2}$ spectra for all 5Ni-$CeO_2$ NCs samples, procedure described in reference [1] was followed. Firstly, as-prepared and lower temperature treated samples, which showed a similar peak shape, was fitted with a combination of NiO and $Ni(OH)_2$. For these compounds, reference [1] provides an empirical fitting in its table 1. Shapes generated with a set of peaks proposed by the authors, reproduce the experimental peak shape obtained for standard samples with known composition. In this case, NiO, $Ni(OH)_2$ and NiOOH were chosen, and binding energy differences, intensity ratios and FWHM ratios for the three phases were reproduced as detailed in ref. [1] Table 1. Figure S2 shows the set of 5 peaks for NiO (blue) and 6 peaks for $Ni(OH)_2$ (red) used to fit experimental data. No data for NiOOH is shown here, as no successful fitting was obtained including this phase. Figure 3 in the main text of this paper shows all peaks blended and filled for each phase, to show more clearly the contribution of NiO and $Ni(OH)_2$ to the experimental spectrum. The same applies to Figure S5.

After reducing at 500 °C, and above this temperature, nickel appears to be reduced. In this case, ref. [1] was used to extract data for metallic nickel fitting. Figure S6 shows the result, with a good fitting of Ni $2p_{3/2}$ using only Ni(0) contribution.

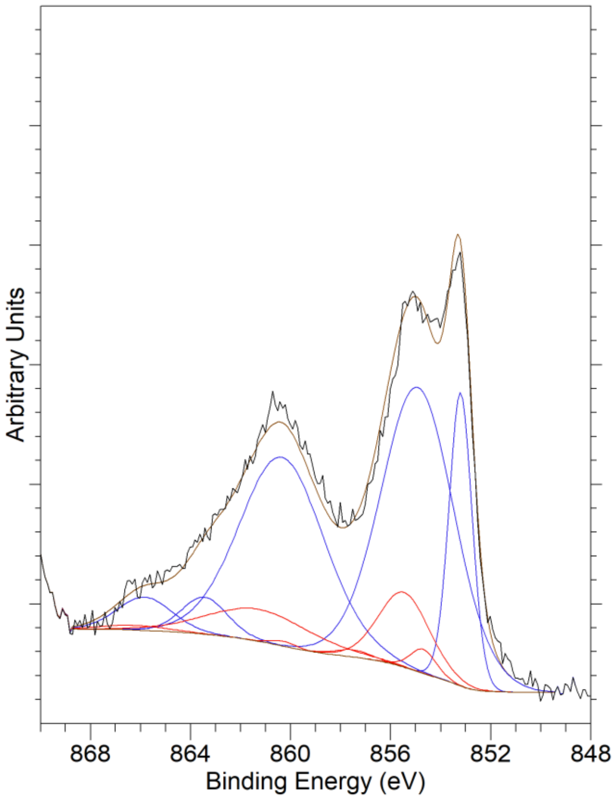


**Figure S2.** Ni $2p_{3/2}$ XPS signal obtained with Al Kα for the as-prepared 5Ni-$CeO_2$ NCs catalyst. Detailed peak decomposition using data from Biesinger et al. [1].

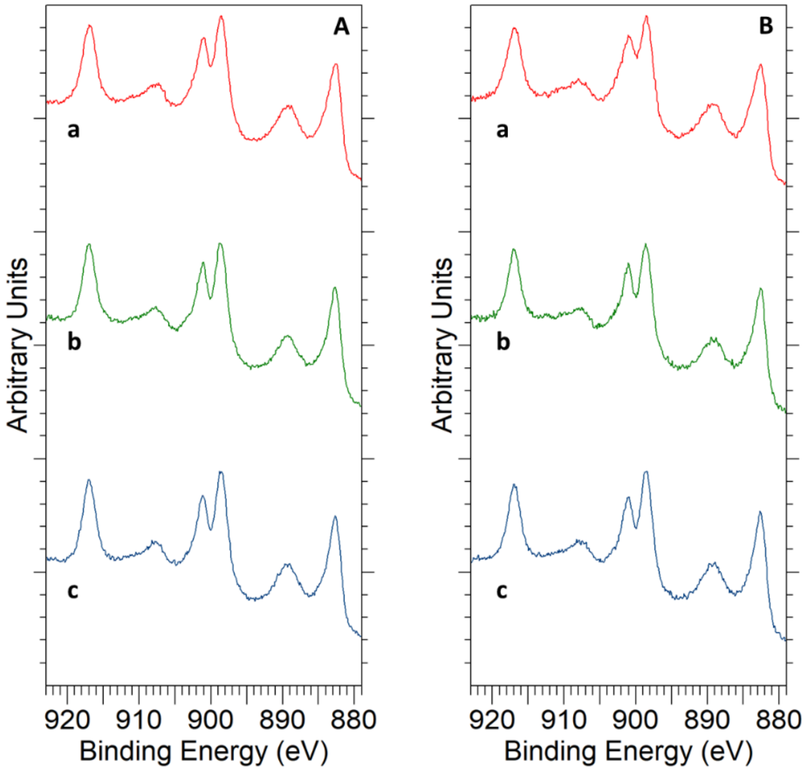


**Figure S3.** Ce 3*d* NAP-XPS spectra recorded at KE = 550 eV (**A**) and KE = 190 eV (**B**) corresponding to 5Ni-$CeO_2$ NCs under different atmospheres at 250 °C: (a) 1 mbar $H_2$, (b) 1 mbar $CO_2$, and (c) 1 mbar $CO_2$ + $H_2$ (1:4).

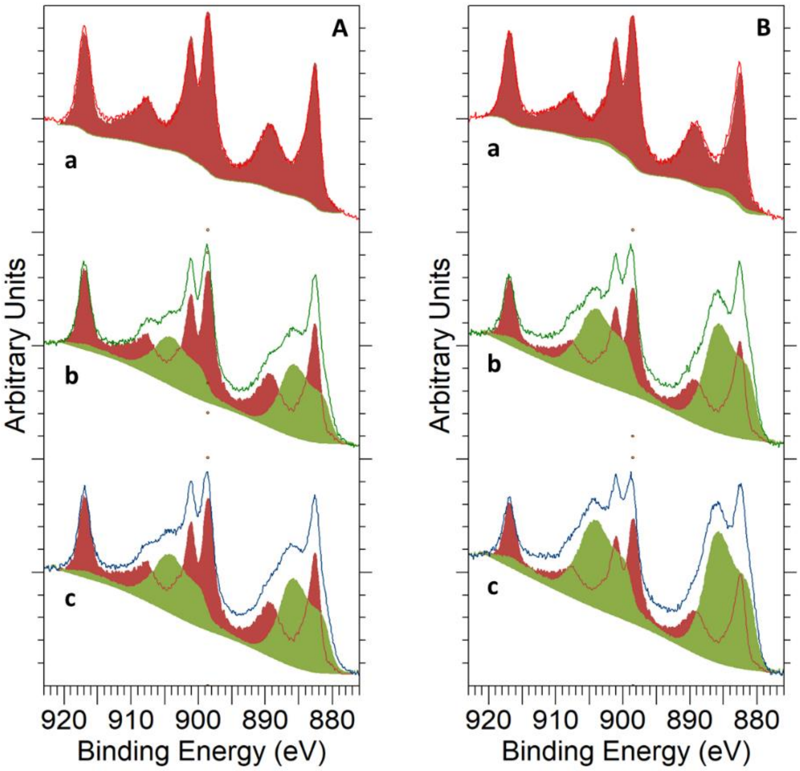


**Figure S4.** Ce 3*d* NAP-XPS spectra recorded at KE = 550 eV (A) and KE = 190 eV (B) corresponding to 5Ni-$CeO_2$ NCs under 1 mbar $CO_2$ + $H_2$ (1:4) at different temperatures: (a) 250 °C, (b) 500 °C, and (c) 600 °C.

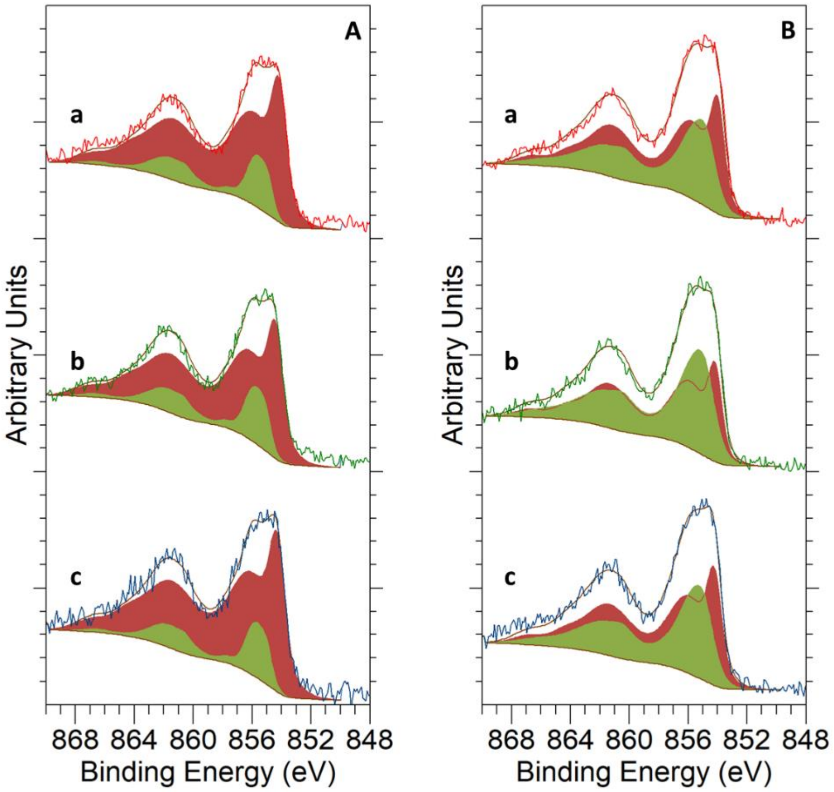


**Figure S5.** Ni 2$p_{3/2}$ NAP-XPS spectra recorded at KE = 550 eV (A) and KE = 190 eV (B) corresponding to 5Ni-$CeO_2$ NCs under 1 mbar $H_2$ at different temperatures: (a) 250 °C, (b) 300 °C, and (c) 350 °C.

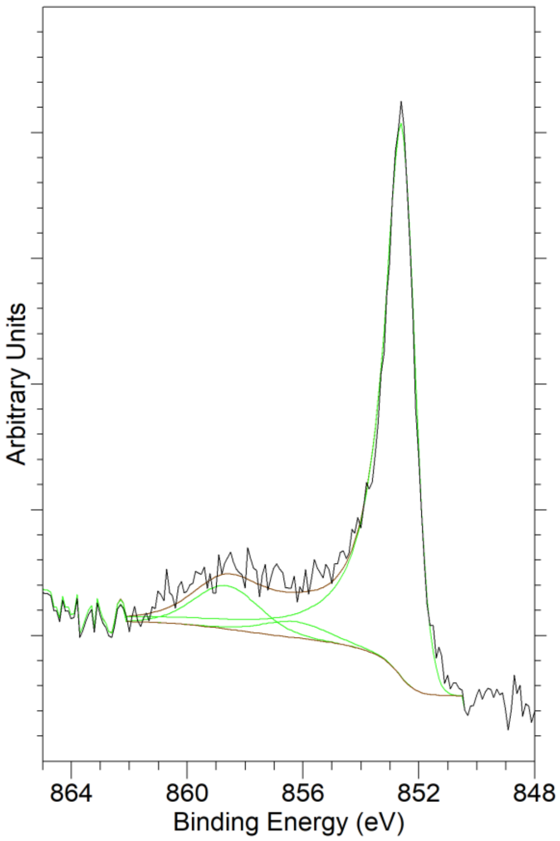


**Figure S6.** Ni $2p_{3/2}$ obtained at KE = 190 eV for 5Ni-$CeO_2$ NCs under 1 mbar $H_2$ at 500 °C.

**C 1s and O 1s Peak Fitting for NAP-XPS Data**

Peak fitting for C 1s and O 1s was performed using mixed Gaussian-Lorentzian (70–30%) lineshapes, and Shirley type backgrounds.

It is known that Ce 4s signal overlaps with C 1s signals, so its contribution to the total spectra had to be taken into account before performing the fittings. First of all, using a clean sample almost free from adventitious carbon or other carbon contributions, C 1s region was adquired to obtain Ce 4s peak shape. The resulting signal corresponds to Ce 4s contribution, so a peak shape could be modeled using this Ce 4s signal. As data was obtained at two different kinetic energies, 550 eV and 190 eV, and two different samples were used, $CeO_2$ NCs and 5Ni-$CeO_2$ NCs, a set of four different lineshapes were obtained for Ce 4s signal, depending on the KE and the sample used. This shape can be seen in dark red in Figures 7–9.

Then, a set of peaks was used for spectral decomposition, using the parameters summarized in Table S1. FWHM for the peaks are not chosen to be the same for different species appearing in the same spectrum. This is due to the different origin of those signals. All signals coming from gas-phase species were set to the same FWHM. These peaks use to be the narrowest peaks in the spectrum. Also, their BEs are linked, so fixed displacements from $CO_2$ are expected for other gas-phase species.

The rest of C 1s signals are fitted by linking their FWHM, and giving a narrow interval for the peaks to move, according to literature [2].

For O 1s, the nature of the signal corresponding to $O^{2-}$ and adsorbed species (including $OH^-$) is different, so FWHM corresponding to $O^{2-}$ was not linked to them. Finally, all adsorbed O 1s species (including $OH^-$) were set to the same FWHM, and given a limited margin to move [2].

**Table S1.** C 1s and O 1s peak fitting parameters.

| Species | | C 1s | | O 1s | |
|---|---|---|---|---|---|
| | | BE (eV) | FWHM (eV) | BE (eV) | FWHM (eV) |
| Gas phase* | $CO_2$ ** | 293.1 ± 0.5 | 0.5 ± 0.1 | 536.5 ± 0.8 | 0.7 ± 0.1 |
| | CO | BE($CO_2$)-1.5 | 0.5 ± 0.1 | BE($CO_2$)-1.38 | 0.7 ± 0.1 |
| | $CH_4$ | BE($CO_2$)-6.5 | 0.5 ± 0.1 | - | - |
| | $H_2O$ | - | - | BE($CO_2$)+1.3 | 0.7 ± 0.1 |
| Structural and adventitious species | $O^{2-}$ § | - | - | 529.7 ± 0.3 | 1.1 ± 0.1 |
| | $OH^-$ | - | - | 530.3 ± 0.2 | 1.6 ± 0.2 |
| | $CH_x$ C–C† | 284.9 ± 0.1 | 1.3 ± 0.2 | - | - |
| | C–O† | 286.2 ± 0.2 | 1.3 ± 0.2 | n.a. † | n.a. † |
| Adsorbed species | $CO_2^{\delta-}$ | 288.0 ± 0.2 | 1.3 ± 0.2 | 531.6 ± 0.3 | 1.6 ± 0.2 |
| | $HCOO^-$ | 289.1 ± 0.2 | 1.3 ± 0.2 | 532.7 ± 0.1 | 1.6 ± 0.2 |
| | $CO_3^{2-}$ | 290.3 ± 0.3 | 1.3 ± 0.2 | 532.1 ± 0.2 | 1.6 ± 0.2 |

Notes: * Signals corresponding to gas phase reaction products are given with respect to gaseous $CO_2$ signal position; ** Significant variability in peak positions for gas phase signals are due to charging effects on the sample. Although charging effects were small enough to avoid significant peak broadening and distortion, it however produced a shift for signals coming from the samples and the adsorbed species. Correction of BE scale was done to shift sample peaks in correct positions, so gas phase signals were artificially shifted due to this correction, resulting in the observed variability in their positions, as they were not affected by sample charging; § $O^{2-}$ signals should include contributions from $CeO_2$ and NiO, that cannot be distinguished; † Signals from adventitious carbon were too weak in most of the cases, so in O 1s core levels could not be detected.

## References


1. Biesinger, M.C.; Payne, B.P.; Lau, L.W.M.; Gerson, A.; Smart, R.S.C. X-ray photoelectron spectroscopic chemical state quantification of mixed nickel metal, oxide and hydroxide systems. *Surf. Interface Anal.* **2009**, *41*, 324–332, doi:10.1002/sia.3026.
2. Zhong, L.; Chen, D.; Zafeiratos, S. A mini review of in situ near-ambient pressure XPS studies on non-noble, late transition metal catalysts. *Catal. Sci. Technol.* **2019**, *9*, 3851–3867, doi:10.1039/c9cy00632j.
